# Composite of KLVFF-Transthyretin-Penetratin and Manganese Dioxide Nanoclusters: A Multifunctional Agent against Alzheimer’s β-Amyloid Fibrillogenesis

**DOI:** 10.3390/molecules29061405

**Published:** 2024-03-21

**Authors:** Haitao Lan, Ying Wang, Wei Liu, Xiaoyan Dong, Yan Sun

**Affiliations:** 1 Key Laboratory of Systems Bioengineering and Frontiers Science Center for Synthetic Biology (Ministry of Education), Department of Biochemical Engineering, School of Chemical Engineering and Technology, Tianjin University, Tianjin 300350, China; lanhaitao@tju.edu.cn (H.L.); 1020207109@tju.edu.cn (Y.W.); d_xy@tju.edu.cn (X.D.); 2 Tianjin Key Laboratory of Radiation Medicine and Molecular Nuclear Medicine, Institute of Radiation Medicine, Chinese Academy of Medical Sciences and Peking Union Medical College, Tianjin 300192, China

**Keywords:** Alzheimer’s disease, amyloid β-protein, transthyretin, manganese dioxide nanoclusters, BBB penetration

## Abstract

Design of amyloid β-protein (Aβ) inhibitors is considered an effective strategy for the prevention and treatment of Alzheimer’s disease (AD). However, the limited blood–brain barrier (BBB) penetration and poor Aβ-targeting capability restricts the therapeutic efficiency of candidate drugs. Herein, we have proposed to engineer transthyretin (TTR) by fusion of the Aβ-targeting peptide KLVFF and cell-penetrating peptide Penetratin to TTR, and derived a fusion protein, KLVFF-TTR-Penetratin (KTP). Moreover, to introduce the scavenging activity for reactive oxygen species (ROS), a nanocomposite of KTP and manganese dioxide nanoclusters (KTP@MnO_2_) was fabricated by biomineralization. Results revealed that KTP@MnO_2_ demonstrated significantly enhanced inhibition on Aβ aggregation as compared to TTR. The inhibitory effect was increased from 18%, 33%, and 49% (10, 25, and 50 μg/mL TTR, respectively) to 52%, 81%, and 100% (10, 25, and 50 μg/mL KTP@MnO_2_). In addition, KTP@MnO_2_ could penetrate the BBB and target amyloid plaques. Moreover, multiple ROS, including hydroxyl radicals, superoxide radicals, hydrogen peroxide, and Aβ-induced-ROS, which cannot be scavenged by TTR, were scavenged by KTP@MnO_2_, thus resulting in the mitigation of cellular oxidative damages. More importantly, cell culture and in vivo experiments with AD nematodes indicated that KTP@MnO_2_ at 50 μg/mL increased the viability of Aβ-treated cells from 66% to more than 95%, and completely cleared amyloid plaques in AD nematodes and extended their lifespan by 7 d. Overall, despite critical aspects such as the stability, metabolic distribution, long-term biotoxicity, and immunogenicity of the nanocomposites in mammalian models remaining to be investigated, this work has demonstrated the multifunctionality of KTP@MnO_2_ for targeting Aβ in vivo, and provided new insights into the design of multifunctional nanocomposites of protein–metal clusters against AD.

## 1. Introduction

Alzheimer’s disease (AD) stands as one of the most prevalent neurodegenerative diseases, with clinical characteristics of memory deficit, cognition impairment, communicative decline, emotional disorder, and disabling behavior. It is a complex and multifactorial disease with pathological mechanisms such as amyloid β-protein (Aβ) aggregation, tau protein hyperphosphorylation, neuroinflammation, and synaptic dysfunction [1,2]. One of the pathologic features of AD is extracellular amyloid plaque deposition, resulting from the gradual accumulation and aggregation of amyloid β-protein (Aβ) in the cerebrum [3]. The amyloid cascade hypothesis suggests that aggregation of Aβ monomers into oligomers and mature fibrils is a major factor contributing to the pathologic dysfunction of neurons [4,5]. Therefore, the exploration of inhibition of Aβ aggregation is considered integral to the prevention and treatment of AD [6,7]. However, accurate delivery of drugs to the lesion site deeply seated in the brain requires drugs to pass through the blood–brain barrier (BBB), which is a major obstacle for AD treatment [8,9]. In the AD patient’s brain, Aβ can interact with corresponding receptors to activate downstream pathways, leading to the production of reactive oxygen species (ROS). Meanwhile, due to the imbalance of metal ion homeostasis, transition metal ions such as Cu^2+^, Zn^2+^, and Fe^3+^ can produce ROS through Fenton-like reactions [10,11,12,13,14]. These excess ROS can lead to mitochondrial dysfunction, resulting in oxidative stress. Therefore, the design of Aβ inhibitors with multifunctionality including BBB penetration and ROS scavenging capability is of great significance for the treatment of AD.

Currently, a variety of Aβ inhibitors have been reported, including biomacromolecules (e.g., peptides and proteins), small molecules (e.g., epigallocatechin gallate and curcumin), and functional nanoparticles (e.g., polymer nanoparticles, gold nanoparticles, and carbon dots) [15,16,17,18,19,20,21]. Of those, protein-based inhibitors have attracted increasing attention because of their favorable biocompatibility, facile modification, and mature methodology [22,23]. Several proteins existing in plasma and cerebrospinal fluid have been validated for their potential to bind to Aβ and interfere with the aggregation [24,25,26,27]. Among them, transthyretin (TTR) has demonstrated efficacy in inhibiting Aβ fibrillogenesis. TTR, initially identified as prealbumin, serves as a principal transport protein for the delivery of thyroxine and retinol and exists as a symmetric tetrameric complex (~55 kDa) [28,29]. It has been demonstrated that TTR can bind to various Aβ species; monomeric TTR tends to bind to Aβ monomers, while tetrameric TTR shows preferential interaction with Aβ aggregates [25]. Besides, previous investigations indicated that TTR monomers showed higher affinity for Aβ than tetramers, probably due to the buried hydrophobic inner sheet of TTR tetramers [30]. Furthermore, Ghadami et al. found that TTR can inhibit primary and secondary nucleation of Aβ and alleviate Aβ-mediated cytotoxicity [31]. However, the inhibition capability of TTR is lower than basified human serum albumin [32], and its low BBB penetration and lack of ROS scavenging activity disallowed it from being a qualified AD-targeting drug. Therefore, it needs to be formulated for better applications.

Cell-penetrating peptides (CPPs) have been widely used for improving intracellular delivery and BBB penetration efficiency [33,34]. Currently, CPPs can be categorized into three types: cationic, amphiphilic, and hydrophobic CPPs [35]. Cationic CPPs typically consist of less than 30 amino acid residues that can help deliver micro- and macromolecules into living cells. Various endocytic pathways coexist during the cellular internalization, including endocytosis, direct translocation, and pathways regulated by clathrin, caveolin, and flotillin [36]. The penetration efficiency of cationic CPPs is intricately linked to the cationic residues, which would interact with the negatively charged moieties of proteoglycans on the cellular membrane [37]. Penetratin (Pen) is a representative cationic CPP derived from the *Antennapedia homeodomain* with the amino acid sequence of RQIKIWFQNRRMKWKK [36,38]. Although Pen-functionalized nanoparticles can cross the BBB, the nanoparticles still hardly accumulated in the lesion site due to the lack of targeting capability. As for delivery of Aβ inhibitors, KLVFF from the central hydrophobic core (CHC) region of Aβ can serve as an Aβ-targeting peptide [39]. For instance, Plissonneau et al. grafted KLVFF onto nanoparticles, thereby conferring upon them the ability to recognize and bind to amyloid plaques in the mouse hippocampus [40]. Thus, the incorporation of Pen and KLVFF in a therapeutic agent is anticipated to augment targeted delivery to amyloid plaques in the brain, thereby enhancing its efficacy.

Recently, various substances with notable ROS scavenging activity have been identified, such as manganese-, carbon-, molybdenum-, and cerium-based nanomaterials [41,42,43,44]. Manganese dioxide (MnO_2_) has attracted broad attention in the biomedical field owing to its excellent catalytic efficiency, robust stability, and facile preparation process [45,46,47]. MnO_2_ exhibits multienzyme activities, including intrinsic superoxide dismutase, peroxidase, and catalase-like enzyme activities. These properties enable it to effectively scavenge harmful ROS within cells. For example, Gao et al. synthesized HSA-MnO_2_ nanocomposites (HMn NCs) through a one-step biomineralization process, which exhibited considerable potential in efficiently scavenging multiple intracellular ROS [48]. Therefore, the combination of protein and MnO_2_ emerged as one of the sensible choices for conferring ROS scavenging activity to AD therapeutic agents.

To address the challenges of low BBB penetration, Aβ-targeting capability, and ROS scavenging activity of TTR, this work is proposed to synthesize MnO_2_ nanoclusters within multifunctional fusion proteins for efficient clearance of amyloid plaques and ROS. Herein, KLVFF-TTR-Pen (KTP) was firstly constructed by fusing TTR with KLVFF at the N-terminal and Pen at the C-terminal. On the one hand, BBB penetration and Aβ-targeting can be introduced into this system. On the other hand, it is expected that the hydrophobicity of KLVFF and Pen may lead to an enhanced inhibitory efficiency of KTP. After that, in order to incorporate ROS scavenging activity, KTP@MnO_2_ (a nanocomposite of KTP and MnO_2_ nanoclusters) was fabricated via biomineralization. The inhibitory effects of KTP@MnO_2_, KTP, and TTR against Aβ aggregation were investigated. Then, an array of experiments was performed to evaluate the BBB penetration, Aβ-targeting, and ROS scavenging activities of KTP@MnO_2_. Finally, the therapeutic effects of KTP@MnO_2_ were explored through cellular and in vivo experiments to broaden the applications of protein and metal-based nanocluster complexes in inhibiting Aβ aggregation.

## 2. Results and Discussion

### 2.1. Protein Purification and Characteristics of KTP@MnO_2_

**Figure 1:**
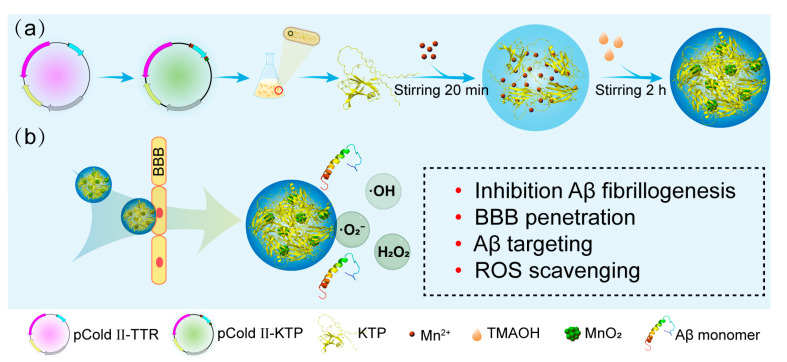
(**a**) Schematic representation of the synthesis of KTP@MnO_2_. (**b**) Multifunctional KTP@MnO_2_: Inhibition Aβ fibrillogenesis, BBB penetration, Aβ targeting, and ROS scavenging.

**Figure 2:**
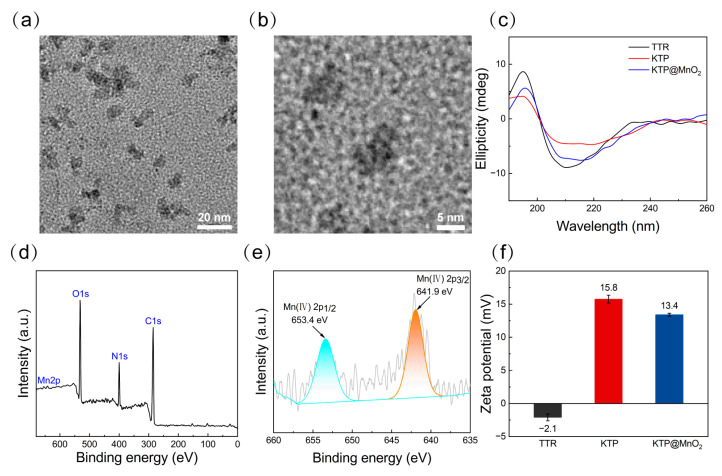
Characteristics of various inhibitors. (**a**) TEM image and (**b**) enlarged image of KTP@MnO_2_. (**c**) CD spectra of TTR, KTP, and KTP@MnO_2_, (**d**) XPS energy spectrum, and (**e**) High-resolution Mn2p XPS spectrum of KTP@MnO_2_. (**f**) ζ-potential of TTR, KTP, and KTP@MnO_2_.

KTP was constructed by fusing TTR with KLVFF at the N-terminal and Pen at the C-terminal. KTP was chosen for subsequent experiments based on pre-experimental findings, demonstrating its superior efficacy compared to KLVFF-TTR in inhibiting Aβ aggregation and theoretical considerations, suggesting that KTP possesses the ability to target Aβ in a manner not achievable by TTR-Penetratin (Figure S1). For the expression of TTR and KTP protein, recombinant vectors pCold II-TTR and pCold II-KTP were constructed and transformed into the *Escherichia coli* BL21 (Figure S2a,b). The purified TTR was composed of monomers (~15 kDa) and dimers (~35 kDa) (Figure S2c), and KTP contained monomers (~20 kDa) and dimers (~45 kDa) (Figure S2d). It has been demonstrated that both TTR monomers and dimers could bind to Aβ species [49], therefore, proteins utilized in the following fabrication experiments were the mixtures of monomers and dimers. As shown in Figure 1, KTP solution was first mixed with manganese chloride (MnCl_2_), and then the composites of KTP and MnO_2_ nanoclusters were formed by a biomineralization process under alkaline conditions [44,50], which were designated as KTP@MnO_2_. KTP@MnO_2_ was relatively uniform with an average size of approximately 8 nm (Figure 2a), and it contained several MnO_2_ nanoclusters with a size of about 1 nm (Figure 2b). The conformations of different inhibitors were determined by CD spectroscopy (Figure 2c). TTR presented β-sheet conformation with a negative peak near 216 nm and a positive peak before 200 nm, which is consistent with the literature [51]. KTP presented α-helix conformation with double negative peaks at 220 nm and 208 nm, and a positive peak before 200 nm. KTP@MnO_2_ maintained a conformation similar to KTP, but the content of secondary structures changed. The increase in the negative ellipticity of KTP@MnO_2_ around 220 nm and 208 nm indicated the increase in α-helix content. The elemental composition and valence distribution were investigated by XPS survey (Figure 2d,e). The XPS full scan spectrum showed that KTP@MnO_2_ was mainly composed of C, N, O, and Mn elements (Figure 2d). Moreover, the high-resolution Mn2p XPS spectra of KTP@MnO_2_ showed two peaks at 641.9 and 653.4 eV (Figure 2e), which corresponded to the characteristic peaks of Mn2p in MnO_2_, indicating the successful synthesis of KTP@MnO_2_. The ζ-potential of TTR was −2.1 mV, and it was increased to 15.8 mV for KTP (Figure 2f) due to the introduction of positively charged KLVFF and Pen [38,52]. Notably, due to the negatively charged MnO_2_, the ζ-potential of KTP@MnO_2_ decreased to 13.4 mV [53].

### 2.2. Inhibition of Aβ_40_ Fibrillization

**Figure 3:**
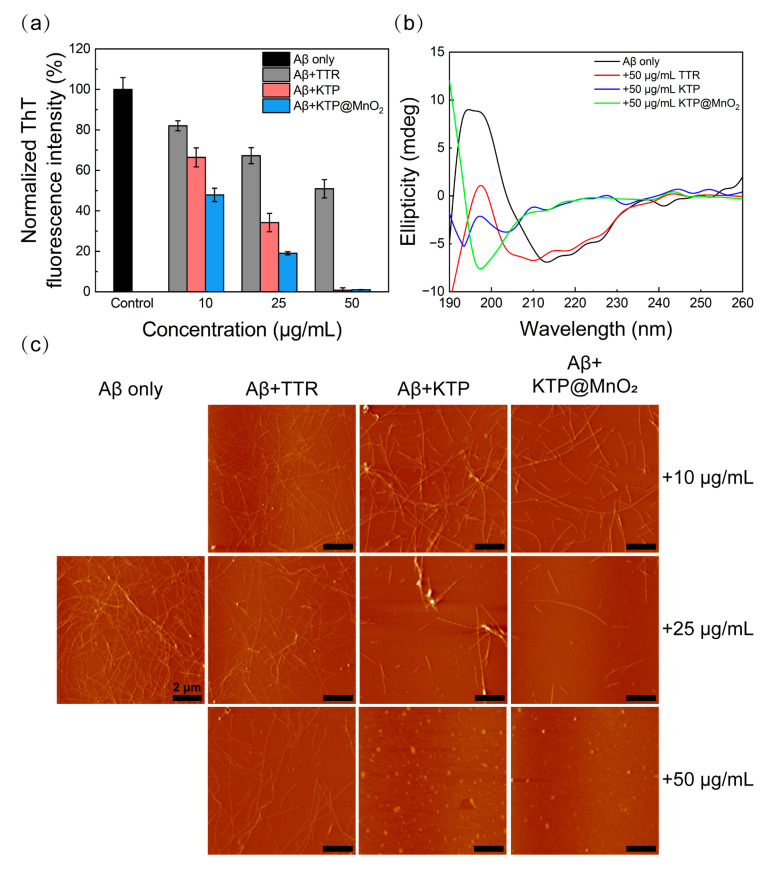
Inhibition of Aβ_40_ aggregation. (**a**) Normalized ThT fluorescence intensity of Aβ_40_ incubated with different concentrations of inhibitors at 160 h. (**b**) CD spectra of Aβ_40_ treated with inhibitor (50 μg/mL) at 160 h. (**c**) AFM images of Aβ_40_ incubated with different concentrations of inhibitors at 160 h. Scale bars are 2 μm.

A ThT fluorescence assay was employed to examine the inhibitory efficiency of various inhibitors, since ThT dyes can bind to the β-sheet structure of Aβ species and exhibit enhanced fluorescence signal. As observed in the aggregation kinetics of Aβ_40_ (Figure S3), Aβ_40_ fibrillization exhibited a sigmoidal curve with a distinct lag period (nucleation phase). Normalized ThT fluorescence intensity after 160 h co-incubation obtained from the kinetic curve of Aβ_40_ in Figure S3 was presented in Figure 3a. TTR, KTP, and KTP@MnO_2_ inhibited Aβ_40_ fibrillization in a concentration-dependent manner, as evidenced by the decreased ThT fluorescence intensity in the plateau phase. TTR inhibited the 18%, 33%, and 49% ThT fluorescence intensity of Aβ_40_ at 10, 25, and 50 μg/mL, respectively. In comparison, KTP possessed a stronger inhibitory capability than TTR, reducing ThT fluorescence intensity by approximately 34%, 66%, and 100% at 10, 25, and 50 μg/mL, respectively. Remarkably, KTP@MnO_2_ showed the most potent inhibitory capability, completely inhibiting the aggregation of Aβ_40_ at 50 μg/mL, and suppressing about 52% and 81% ThT fluorescence intensity at 10 and 50 μg/mL. The inhibitory capability of KTP@MnO_2_ was significantly stronger than that of human serum albumin (HSA), an Aβ-binding protein, which reduced ThT fluorescence intensity by 20% at 0.5 μM (33.3 μg/mL). Furthermore, KTP@MnO_2_ demonstrated greater inhibition compared to the nanocomposite formed by the combination of HSA and MnO_2_ (25 μg/mL and 50 μg/mL of HSA@MnO_2_ inhibited 64% and 72% of ThT fluorescence intensity, respectively) [48]. It is considered that the high inhibitory capability of KTP was attributed to the introduction of positively charged and hydrophobic KLVFF and Pen, which enhanced the electrostatic and hydrophobic interactions between KTP and Aβ_40_. This inference was corroborated by isothermal titration calorimetry assay (Figure S4). The Δ*G* values for the binding of TTR and KTP to Aβ_40_ were determined to be −36.83 and −48.43 kJ/mol, respectively, signifying the spontaneous nature of their binding events with Aβ_40_. Furthermore, the Δ*G* and *K*_d_ values between KTP and Aβ_40_ were calculated to be smaller than those of TTR, suggesting a stronger binding affinity between KTP and Aβ_40_. In addition, it is considered that the introduction of MnO_2_ nanoclusters could stabilize the conformation of KTP, resulting in robust inhibitory capability. The lag time (*T*_lag_) obtained from the kinetic curve of Aβ_40_ in Figure S3 is listed in Table S1. It can be seen that *T*_lag_ increased with the addition of TTR, which was attributed to TTR being able to affect the primary nucleation and secondary nucleation of Aβ_40_ by binding to Aβ_40_ oligomers [31]. *T*_lag_ became shorter with the addition of KTP, indicating KTP could accelerate nucleation and promote the early aggregation of Aβ_40_ [32,54]. However, the *T*_lag_ of KTP@MnO_2_ was longer than that of KTP, suggesting that the introduction of MnO_2_ can extend the *T*_lag_ [48].

To get insight into the conformational transition of Aβ_40_, CD spectroscopy was performed (Figure 3b and Figure S5). After 160 h of incubation, Aβ_40_ formed a typical β-sheet structure with negative peak at 216 nm and positive peak near 195 nm (Figure 3b, black line). The ellipticity value of Aβ_40_ changed with the addition of KTP or KTP@MnO_2_. Significantly, with 50 μg/mL of KTP or KTP@MnO_2_, the typical β-sheet structure disappeared, indicating that the aggregation of Aβ_40_ was completely inhibited. The BeStSeL algorithm was employed to evaluate the impact of the inhibitor on the secondary structure compositions of Aβ_40_. As illustrated in Table S2, helix and antiparallel β-sheet structures coexisted in Aβ_40_ before incubation, whereas antiparallel β-sheets decreased and parallel β-sheets increased after incubation. The reason for the change in secondary structure from antiparallel to parallel β-sheets is that antiparallel β-sheets could cause instability in the salt bridge within Aβ, which promotes the aggregation of Aβ_40_ to form mature fibrils with lower energy [55,56,57]. The content of parallel β-sheet structures of Aβ_40_ decreased slightly after co-incubation with TTR. Notably, co-incubation with KTP or KTP@MnO_2_ led to the complete elimination of parallel β-sheet structures in Aβ_40_. This result demonstrated that inhibitors may have altered the aggregation pathway of Aβ_40_, which no longer aggregated into fibrils. Moreover, the morphology of Aβ_40_ aggregates co-incubated with inhibitors was observed by AFM (Figure 3c). Aβ_40_ aggregated into dense, elongated, and intertwined fibrils. The number and length of Aβ_40_ fibrils decreased with increasing concentrations of inhibitors, and at the same concentration, Aβ_40_ co-cultured with KTP@MnO_2_ contained the fewest fibrils. Fibrils were observed in Aβ_40_ co-incubated with 50 μg/mL TTR, whereas fibrils disappeared in Aβ_40_ co-incubated with 50 μg/mL KTP and KTP@MnO_2_. Taken together, CD spectroscopy and AFM imaging further verified the results of ThT fluorescence experiments. KTP@MnO_2_ could effectively inhibit Aβ_40_ aggregation. Due to the introduction of the Aβ-targeting peptide-KLVFF and BBB-penetrating peptide-Pen into KTP@MnO_2_, it is expected to possess the functionalities of Aβ targeting and BBB penetration. Therefore, the BBB penetration and Aβ targeting of KTP@MnO_2_ will be investigated in detail next.

### 2.3. BBB Penetration

**Figure 4:**
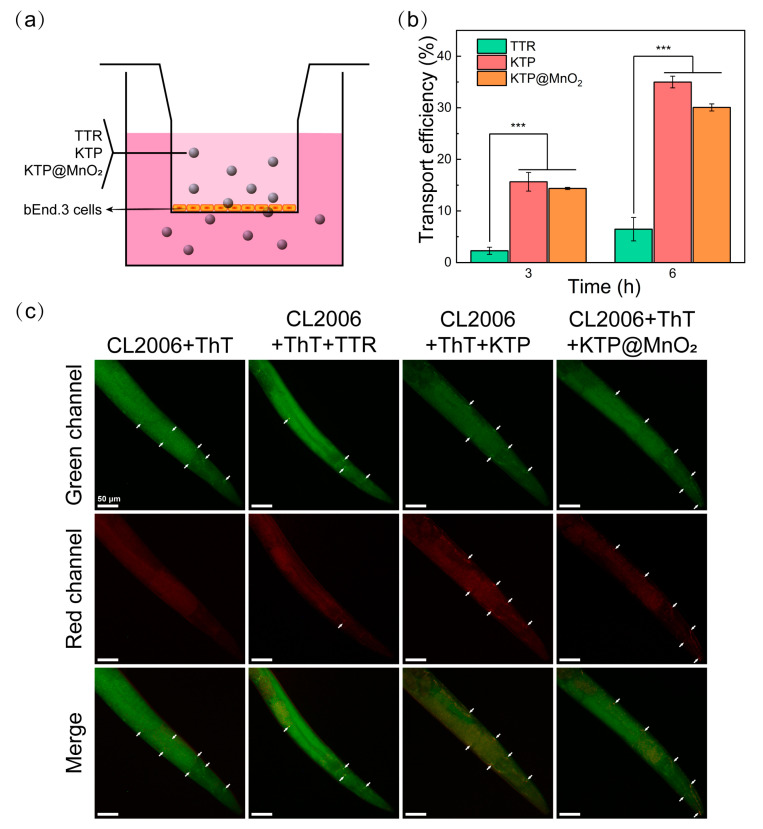
(**a**) Schematic representation of inhibitors crossing the BBB model. (**b**) Quantitative analysis of the BBB penetration efficiency of TTR, KTP, and KTP@MnO_2_. Statistical significance level was expressed by asterisk (in comparison with the TTR group, *** *p* < 0.001). (**c**) In vivo targeting capability of different inhibitors. *C. elegans* were stained with Aβ-specific probe ThT (green emission) and incubated with Cy5-labelled inhibitors (red emission). Scale bars are 50 μm. The amyloid plaques were marked by white arrows.

The feasibility of Pen to enhance the BBB penetration of inhibitors was assessed through Transwell experiments [58,59,60,61]. As illustrated in Figure 4a, a tightly connected monolayer of cell membranes was formed by inoculation of bEnd.3 cells onto the Transwell membrane, and Cy5-labelled TTR, KTP, and KTP@MnO_2_ were added to the upper chamber, individually. Subsequently, the solution in the lower chamber was collected for quantitative analysis of BBB penetration at 3 h and 6 h. The linear correlation between the concentration and fluorescence intensity is established in Figure S6, and the quantitative BBB penetration ratio was calculated by measuring the fluorescence intensity in the lower chamber (Figure 4b). The penetration efficiencies of TTR were 2.3% and 6.5% at 3 and 6 h, respectively. In contrast, the penetration efficiency of KTP was significantly higher, reaching 15.7% and 35.0% at 3 and 6 h. KTP@MnO_2_ showed a slight decrease in penetration efficiency compared to KTP, with penetration efficiencies of 14.4% and 30.1% at 3h and 6 h. This slight decline in penetration efficiencies may be attributed to the larger particle size of KTP@MnO_2_. Significantly, the penetration efficiency of KTP@MnO_2_ surpasses that of nanomedicine modified by the brain-targeting peptide RVG (25% penetration efficiency after 10 h in the same model) [58], and aligns closely with that of another Pen-modified protein agent (17.5% penetration efficiency after 3 h and 31.1% after 6 h with the same model) [32]. Overall, the elevated BBB penetration of KTP@MnO_2_ can be ascribed to the synergistic effects of Pen and KLVFF, wherein the positive charge and hydrophobicity (lipophilicity) play pivotal roles in the penetration and internalization processes. The above results demonstrated the great potential of KTP@MnO_2_ to penetrate the BBB and be utilized as an anti-Aβ aggregation agent.

### 2.4. Targeting Amyloid Plaques in C. elegans

To validate Aβ plaques targeting capability of different inhibitors, the Aβ plaques were stained with ThT and incubated with Cy5-labelled inhibitor (TTR, KTP, or KTP@MnO_2_). As depicted in Figure S7, Aβ exhibited green fluorescent plaques, and a small amount of red fluorescence can be observed in the green regions, indicating that TTR possessed weak binding ability with Aβ plaques. Remarkably, the effective colocalization phenomenon of green-on-red fluorescence for KTP and KTP@MnO_2_ samples indicated that KTP and KTP@MnO_2_ had a higher binding ability with Aβ plaques.

CL2006 nematodes, an AD mutant nematode capable of expressing Aβ_42_ in the muscle of the body wall, were subjected to targeting experiments. As demonstrated in Figure 4c, CL2006 nematodes incubated with Cy5-labelled KTP or Cy5-labelled KTP@MnO_2_ not only exhibited green fluorescence of ThT, but also displayed red fluorescence of Cy5 at the corresponding positions. However, in CL2006 nematodes incubated with Cy5-labelled TTR, predominantly only green fluorescence was observed. The above results suggested that KTP and KTP@MnO_2_ possessed the ability to target amyloid plaques in CL2006 nematodes.

### 2.5. ROS Scavenging Activity

**Figure 5:**
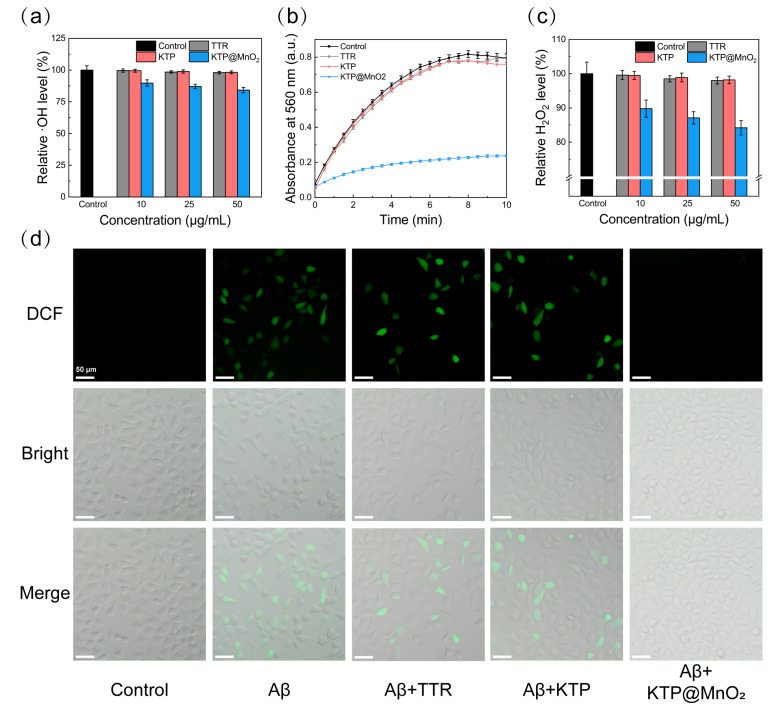
ROS scavenging ability of different inhibitors. (**a**) ·OH, (**b**) ·O_2_^−^, and (**c**) H_2_O_2_ scavenging abilities of different inhibitors. (the concentration of inhibitor was 50 μg/mL). (**d**) Detection of Aβ-induced ROS in SH-SY5Y cells by fluorescent microscopy. The concentration of Aβ was 25 μM. The concentration of inhibitor was 50 μg/mL. Scale bars are 50 μm.

·OH can oxidize 2-deoxy-D-ribose to form malondialdehyde, which reacts with TBA to generate 3,5,5-trimethyloxazole-2,4-dione with a strong absorption at 532 nm. The scavenging activity of inhibitors against ·OH was examined by measuring their inhibitory efficiency on 2-deoxy-D-ribose oxidation [62]. As shown in Figure S8, the absorbance did not change after the addition of TTR or KTP. In contrast, the addition of KTP@MnO_2_ led to a notable decrease in the absorbance, indicating that KTP@MnO_2_ has ·OH scavenging activity. KTP@MnO_2_ scavenged approximately 45% and 60% of ·OH at 10 and 50 μg/mL (Figure 5a), which is consistent with the fact that high-valent manganese induces the conversion of ·OH to water and molecular oxygen [63].

·O_2_^−^ can catalytically reduce NBT to form formazan with maximum absorption at 560 nm. Therefore, the ·O_2_^−^ scavenging activity of inhibitors was determined by assessing the inhibition on formazan formation [64,65,66]. As demonstrated in Figure 5b, the absorbance of the control group increased with the duration of illumination, implying the production of ·O_2_^−^. In comparison to the control group, TTR or KTP induced slight changes in absorbance. By contrast, KTP@MnO_2_ significantly reduced the absorbance to 30% at 10 min, demonstrating a superior ·O_2_^−^ scavenging efficiency. Two main reasons can be responsible for the·O_2_^−^ scavenging activity of KTP@MnO_2_. Firstly, MnO_2_ can catalyze the disproportionation reaction of ·O_2_^−^ to generate water and molecular oxygen [46]. Secondly, Mn^2+^ can be produced during the catalytic process, and can chelate with phosphate ligands in the physiological environment, thereby enhancing the disproportionation of·O_2_^−^ [47,67,68].

Moreover, H_2_O_2_ was found to be scavenged by KTP@MnO_2_ in vitro. As depicted in Figure 5c, KTP@MnO_2_ at 50 μg/mL reduced the H_2_O_2_ level by more than 15%, whereas TTR and KTP lowered the H_2_O_2_ level by less than 3%, highlighting the superior H_2_O_2_ scavenging activity of KTP@MnO_2_. Overall, the in vitro ROS scavenging activity of KTP@MnO_2_ was satisfactory, with the ability to eliminate ·OH, ·O_2_^−^, and H_2_O_2_, holding the promise to scavenge free radicals in the protein aggregation process.

DCFH-DA was used to detect intracellular ROS levels. The intensity and distribution of green fluorescence were measured to evaluate ROS levels in cells. After incubation with Aβ_40_, cells showed prominent green fluorescence, whereas cells in the control group displayed no green fluorescence, indicating that Aβ_40_ induced the substantial production of ROS in the cells (Figure 5d). TTR and KTP partially mitigated Aβ_40_-induced ROS, but distinct green fluorescence was still observed. Encouragingly, the addition of KTP@MnO_2_ significantly diminished the green fluorescence, suggesting that KTP@MnO_2_ can effectively eliminate intracellular ROS and mitigate oxidative damage to cells.

Thus, KTP@MnO_2_ with Aβ targeting and inhibition, BBB penetration, and multiple ROS scavenging activities hold exciting promise for efficient and multi-target treatment of AD. The therapeutic effect of KTP@MnO_2_ in cells and nematodes will be showcased in the following section.

### 2.6. Inhibition of Aβ-Induced Cytotoxicity and Scavenging Amyloid Plaques in C. elegans

**Figure 6:**
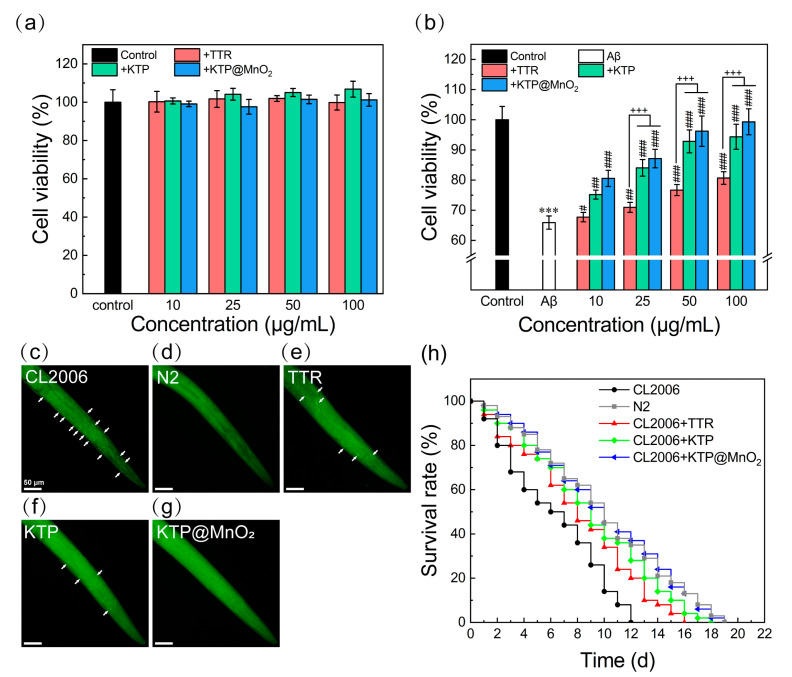
Cell viability and *C. elegans* (N2 and CL2006) assays. (**a**) Viability of SH-SY5Y cells determined by MTT assay. (**b**) The detoxification of different inhibitors on Aβ_40_-induced cytotoxicity. The concentration of Aβ_40_ was 25 μM. Statistical significance level was expressed by asterisk (in comparison with the control group, *** *p* < 0.001), pound sign (in comparison with Aβ group, # *p* < 0.05, ## *p* < 0.01, ### *p* < 0.001) and plus sign (in comparison with TTR group, +++ *p* < 0.001). (**c**–**g**) ThT fluorescence imaging of amyloid plaques in N2 and CL2006 nematodes. CL2006 at L4 stage were incubated with different inhibitors for 3 d. ThT (green emission) was used as an Aβ-specific fluorescence probe to stain all nematodes at the end of incubation. The amyloid plaques were marked by white arrows. Scale bars are 50 μm. (**h**) Survival curves of CL2006 nematodes treated with different inhibitors.

MTT assays were conducted with SH-SY5Y cells to evaluate the detoxification effect. As can be seen from Figure 6a, within the tested concentration range (10–100 μg/mL), inhibitors showed no obvious cytotoxicity, maintaining more than 90% cell viability. When pre-cultured Aβ_40_ was co-cultured with SH-SY5Y cells for 24 h, a notable decrease in cell activity to 66% was observed (Figure 6b). With the increase in the concentration of inhibitors, the cell activity of Aβ_40_ treatment increased. Notably, the cytotoxicity induced by Aβ_40_ was completely inhibited by KTP@MnO_2_ at 50 μg/mL.

The impact of inhibitors on suppressing in vivo Aβ_40_ amyloid plaque formation was investigated using wild-type N2 and AD mutant CL2006 nematodes. After staining with ThT, distinct green fluorescent spots were observed in adult CL2006 nematodes (Figure 6c). As a control, no such fluorescent spots were observed in wild-type N2 nematodes (Figure 6d). CL2006 nematodes were administered with 50 µg/mL of inhibitor at the L4 larval stage and cultured for 3 days before being stained with ThT. The green fluorescent spots in CL2006 nematodes treated with TTR decreased slightly (Figure 6e), and those in CL2006 nematodes treated with KTP decreased significantly (Figure 6f). More importantly, the green fluorescent spots in CL2006 nematodes treated with KTP@MnO_2_ completely disappeared (Figure 6g), indicating that KTP@MnO_2_ at a concentration of 50 μg/mL completely inhibited the deposition of Aβ plaques in CL2006 nematodes. Furthermore, the accumulation of Aβ in CL2006 nematodes could lead to motility impairment and paralysis, which resulted in nematode death within 12 days (Figure 6h). Therefore, the potential of inhibitors to extend the longevity of CL2006 nematodes was evaluated through a lifespan assay. TTR, KTP, and KTP@MnO_2_ prolonged the lifespan of CL2006 nematodes by 4, 6, and 7 days, respectively, and the lifespan of CL2006 nematodes treated with KTP or KTP@MnO_2_ was consistent with that of N2 nematodes. The above results indicate that KTP@MnO_2_ significantly inhibits amyloid aggregation and deposition in vivo, attenuating Aβ-induced toxicity and thereby prolonging the lifespan of CL2006 nematodes.

However, it should be noted that there are several limitations of using *C. elegans* as an AD model. Some AD-related genes such as β-secretase, are deficient in *C. elegans*, which prevents the organism from endogenously producing β-amyloid peptides. Additionally, *C. elegans* lacks many mammalian features, including the circulatory system, myelinated neurons, hippocampus, and adaptive immune system. Therefore, further testing using other models such as zebrafish and mouse models is necessary before considering clinical trials. Furthermore, when applied to the human body, KTP@MnO_2_ may be readily cleared by the hepatobiliary system, and the cationic KTP@MnO_2_ may adsorb negatively charged serum proteins, leading to agglomeration in the circulation. Thus, future studies need to focus on detailed in vivo experiments to test the clinical applicability of nanocomposites. In this paper, the effect of KTP@MnO_2_ on Aβ was investigated, and it may be worthwhile to consider its effect on other pathogenic amyloids (e.g., tau proteins, pancreatic amyloid, α-synuclein, etc.), or to explore its effect on cross-aggregation between different amyloids.

## 3. Materials and Methods

### 3.1. Materials

Synthesized plasmids (pCold II-TTR and pCold II-KTP) were obtained from GENEWIZ (Suzhou, China). Tryptone, yeast extract, and agar powder were purchased from Oxoid (Berkshire, UK). Aβ_40_ (>95%, lyophilized powder) was obtained from GL Biochem (Shanghai, China). Ampicillin (AMP), Isopropyl β-D-Thiogalactoside (IPTG), Thioflavin T (ThT), 1,1,1,3,3,3-hexafluoro-2-propanol (HFIP), 3-(4,5-dimethylthiazol-2-yl)-2,5-diphenyltetrazolium bromide (MTT) were received from Sigma-Aldrich (St. Louis, MO, USA). The reactive oxygen assay kit and catalase assay kit were purchased from Beyotime Biotechnology (Shanghai, China). Fetal bovine serum (FBS), Dulbecco’s modified Eagle’s medium/Ham’s F-12 (DMEM/F12), Dulbecco’s modified Eagle’s medium (DMEM), penicillin-streptomycin were purchased from Gibco (Grand Island, NY, USA). Human neuroblastoma SH-SY5Y cells were from the Cell Bank of the Chinese Academy of Sciences (Shanghai, China). Mouse brain microvascular endothelial cells (bEnd.3) were purchased from Beijing Dingguo Biotechnology Co., Ltd. (Beijing, China). The wild-type N2 strain and the transgenic CL2006 strain of Caenorhabditis elegans (*C. elegans*) were obtained from the Caenorhabditis Genetics Center at the University of Minnesota (Minneapolis, MN, USA). Other chemicals were all of the highest purity and available from local sources.

### 3.2. Protein Expression and Purification

The constructed plasmids mentioned above were transformed into *Escherichia coli* BL21. The strain was cultured overnight for 12 h at 37 °C, 220 rpm using liquid LB medium (1% tryptone, 0.5% yeast powder, 1% NaCl, and 100 μg/mL ampicillin) to obtain the primary seed solution. Fresh prepared LB medium was inoculated with the above seed solution, and cultured at 37 °C, 220 rpm until OD_600_ value reached 0.6~0.8, and then induced with 1 mM IPTG for 4~6 h. The fermentation broth was collected by centrifuging for 30 min at 4 °C, 5000 rpm. After full suspension with lysis buffer (20 mM Tris-HCl, 500 mM NaCl, 20 mM imidazole, pH 8.0) and standing in an ice bath for 30 min, the cells were crushed with an ultrasonic cell crusher (JY92-IIN, Scientz, Ningbo, China) and centrifuged (4 °C, 10,000 rpm, 30 min) to collect the supernatant. To separate the target protein KTP, the supernatant was passed through an Ni affinity chromatography column, followed by removal of nonspecifically adsorbed heterogeneous proteins with washing buffer (20 mM Tris-HCl, 500 mM NaCl, 60 mM imidazole, pH 8.0), and elution with elution buffer (20 mM Tris-HCl, 500 mM NaCl, 500 mM imidazole, pH 8.0). The target proteins were lyophilized and stored after dialysis.

### 3.3. Synthesis and Characterization of KTP@MnO_2_

For synthesis using KTP as template, KTP was mixed with Mn^2+^, and the pH value was adjusted to alkaline with C_4_H_13_NO. The reaction of 2Mn^2+^ + 4OH^−^ + O_2_ → 2MnO_2_ + 2H_2_O was triggered and KTP@MnO_2_ nanoparticles could be obtained by biomineralization. Briefly, MnCl_2_ (0.5 mM, 10 mL) aqueous solution was slowly dropped into protein aqueous solution (1 mg/mL, 10 mL) and incubated for 20 min with vigorous stirring. Then, 50 μL of C_4_H_13_NO (25% aqueous solution) was dropwise added into the reaction solution, followed by vigorous stirring for 2 h to obtain KTP@MnO_2_. The reaction product was dialyzed against deionized water for 2 d (MWCO: 7000 Da), and the insoluble large particles were removed by syringe filter (0.45 μm). Finally, the KTP@MnO_2_ was lyophilized and refrigerated at −20 °C.

The morphology and size distribution of KTP@MnO_2_ were observed by transmission electron microscope (TEM) (JEM-2100F, JEOL, Tokyo, Japan). The conformation of KTP@MnO_2_ was measured by circular dichroism spectrometer (J-180, Jasco, Tokyo, Japan). The element composition and surface chemistry of KTP@MnO_2_ were measured with an X-ray photoelectron spectrometer (XPS) (Kalpha, Thermo Fisher, Waltham, MA, USA) with single X-ray source Al Kα excitation (1486.6 eV). The ζ-potential of KTP@MnO_2_ was measured by a Zetasizer (Nano ZS90, Malvern Panalytical Ltd., Malvern, UK).

### 3.4. Preparation of Aβ Monomer

Aβ_40_ powder was dissolved in HFIP to 1 mg/mL, and ultrasonically treated in an ice bath to destroy the pre-existing Aβ_40_ fibrils. Then it was freeze-dried using a vacuum freeze dryer (Labconco, Kansas City, MO, USA) to obtain lyophilized Aβ_40_. Before use, the treated Aβ_40_ was dissolved in 20 mM NaOH to 275 µM and ultrasonically treated in an ice bath until complete dissolution. The treated solution was centrifuged at 4 °C and 16,000× *g* for 20 min, and then 75% supernatant was carefully collected as Aβ_40_ stock solution for later use.

### 3.5. ThT Fluorescent Assay

ThT can bind to β-sheet rich structures in Aβ aggregates to show enhanced fluorescence intensity (excitation and emission at 440 and 480 nm, respectively). The aggregation kinetics of Aβ_40_ were determined by in situ culture. Aβ_40_ monomer, ThT, and inhibitors with different concentrations were mixed and added to a 96-well plate, in which the final concentration of Aβ_40_ and ThT was 25 μM. Then, the 96-well plate was measured by microplate reader (TECAN Infinite, Salzburg, Austria). The temperature was 37 °C and the measurement time interval was 10 min. The final fluorescence intensity was obtained by subtracting the background fluorescence. The results of the ThT fluorescence experiments were normalized and fitted using sigmoidal Boltzmann curves:

(1) $$y=y_{0}+\frac{y_{\max}-{\;y}_{0}}{1+{\;e}^{-(t\;-{\;t}_{1/2})k}}$$

where *y* is the fluorescence intensity at time t, *y*_0_ and *y*_max_ are the minimum and maximum fluorescence intensity during the aggregation process, respectively, *t*_1/2_ is the corresponding time when the fluorescence value reaches half of the maximum value, and k is the growth rate constant. The lag time (*T*_lag_) was then calculated using the following equation:

(2) $$T_{\mathrm{lag}}=t_{1/2}-\frac{2}{k}$$

### 3.6. Circular Dichroism (CD) Spectroscopy

The effect of inhibitors on the secondary structures of Aβ_40_ aggregates was investigated using a CD spectrometer (J-810, Jasco, Japan). The ellipticity between 190 and 260 nm of Aβ_40_ (25 μM) co-cultured with different concentrations of inhibitors was determined using a 1 mm quartz cell with a bandwidth of 2 nm and a spectral scanning speed of 100 nm/min. Data were averaged over three consecutive scans and the spectrum of inhibitor alone was subtracted from that of the mixture of Aβ with inhibitor.

### 3.7. Atomic Force Microscopy (AFM)

The morphology of Aβ_40_ aggregates was observed in the tap mode of AFM (CSPM5500, Benyuan, Beijing, China). The sample (50 μL) was dropwise added into a freshly peeled clean and flat mica sheet and left for 5 min, followed by rinsing with ultrapure water to remove salt from the samples, and finally dried using a spin coater (KW-A4, IMECAS, Beijing, China) at 1000 rpm for 60 s.

### 3.8. Isothermal Titration Calorimetry (ITC)

An isothermal titration calorimeter (Affinity ITC, TA, New Castle, DE, USA) was employed to ascertain the interaction force between inhibitor and Aβ_40_. The freshly prepared Aβ monomer solution (25 μM) and the inhibitor solution underwent degassing for 10 min. Subsequently, 500 μL of the Aβ monomer solution was introduced into the cuvette, while 100 μL of the inhibitor solution was loaded into the injection needle. The sample underwent titration 25 times at 37 °C, with a titer volume of 2 μL, while the stirring speed was maintained at 100 rpm. The obtained results were adjusted by utilizing the dilution heat of inhibitor titration buffer as a reference background.

### 3.9. In Vitro BBB Transportation Studies

The in vitro BBB model was constructed using bEnd.3 cells according to previous reports [58,69]. bEnd.3 cells (100 cells/μL, 250 μL) were seeded on 24-well Transwell filters (Corning, Glendale, AZ, USA). The transendothelial electrical resistance (TEER), measured with a Millicell-ERS voltohmmeter (Millipore, Sherwood, OR, USA), exceeding 150 Ω × cm^2^ indicated the formation of tightly connected cell layer membrane. Meanwhile, Cy5-labelled inhibitor (0.5 mg/mL, 50 μL) was added to the upper chamber, and PBS buffer (pH 7.4, 1 mL) was used to replace the culture medium in the lower chamber. A 200 μL sample from the lower chamber was collected at 3 h and 6 h, followed by detection of the fluorescence intensity at 710 nm (excitation wavelength 630 nm). Since the fluorescence intensity was linearly related to the concentration, the penetration efficiency was estimated according to the following equation:

(3) $$\mathrm{Penetration}\;\mathrm{efficiency}\left(\%\right)=\frac{100\%\;\times \;(I_{b}-I_{c})}{I_{a}-I_{c}}$$

where *I*_b_ and *I*_a_ are the fluorescence intensity of the lower chamber and the theoretical equilibrium fluorescence intensity of sample, respectively, and *I*_c_ is the background fluorescence intensity of the control group.

### 3.10. In Vitro ROS Scavenging Experiment

Hydroxyl radicals (·OH) were produced by the Fenton reaction of FeSO_4_-H_2_O_2_ [62]. Thiobarbituric acid (TBA) was used to determine the amount of ·OH. ·OH can degrade 2-deoxy-D-ribose to malondialdehyde, which reacts with TBA to produce reddish-brown 3,5,5-trimethyloxazolidine-2,4-dione with the maximum absorption at 532 nm. A quantity of 50 μL of a mixed solution containing ascorbic acid (80 μM), 2-deoxy-D-ribose (20 mM), and FeSO_4_ (80 μM) was added into 100 μL of inhibitor solution. The reaction was initiated by adding 50 μL of H_2_O_2_ (880 μM). After incubation at 37 °C for 90 min, the solution was mixed with TBA (100 μL, 0.4%, *w*/*v*) and trichloroacetic acid solution (100 μL, 6%, *w*/*v*) and boiled for 15 min. After cooling to room temperature and centrifugation, the absorbance of supernatant at 532 nm was measured. The experiment was set up in four parallel groups so as to calculate the mean and standard deviation.

Superoxide radical (·O_2_^−^) was produced by reoxidation of photo-reduced riboflavin under aerobic conditions [64,65]. ·O_2_^−^ can react with nitrobluetetrazolium (NBT) to generate blue formazan with maximum absorption at 560 nm. Briefly, 120 μL of inhibitor solution was configured, followed by the addition of PB buffer (50 mM, pH 7.8, 600 μL), methionine solution (130 mM, 120 μL), nitrobluetetrazolium solution (750 μM, 120 μL), EDTA·Na_2_ solution (100 μM, 120 μL), and riboflavin solution (20 μM, 120 μL). The solutions were mixed and exposed to 30,000 lux of light for several minutes and then the absorbance of the reaction system at 560 nm was measured. The experiment was set up in four parallel groups so as to calculate the mean and standard deviation.

Hydrogen peroxide (H_2_O_2_) scavenging activity was assessed using a catalase assay kit. In brief, inhibitor solution was treated with excess H_2_O_2_ (250 mM) for 5 min at 25 °C. Following the reaction, the remaining H_2_O_2_ was treated with peroxidase to generate the red product, and the absorbance at 520 nm was subsequently measured.

### 3.11. Cell Viability Assay

Cytotoxicity was examined by 3-(4,5-Dimethylthiazol-2-yl)-2,5-diphenyltetrazolium bromide (MTT) assay. SH-SY5Y cells were added to 96-well plates at a density of 8000 cells/well (80 μL) and incubated for 24 h. Subsequently, 20 μL of a mixed solution (containing 25 μM Aβ_40_ and different concentrations of inhibitors) that had been preincubated for 24 h was added, and the incubation was continued for 24 h. After that, MTT solution (10 μL, 5.5 μg/mL in PBS buffer) was added into each well and cultured for 4 h. The 96-well plates were centrifuged at 1500 rpm for 10 min to remove the medium, and then 100 μL of DMSO was added to dissolve and release the bluish-purple formazan crystals. Finally, the absorbance at 570 nm was determined. Six parallels were set up for each set of samples. Wells treated with PBS buffer served as controls. Cell viability was calculated according to the following equation:

(4) $$\mathrm{Cell}\;\mathrm{viability}\left(\%\right)=\frac{100\%\;\times \;({\mathrm{OD}}_{\mathrm{Treated}}-{\mathrm{OD}}_{\mathrm{Background}})}{{\mathrm{OD}}_{\mathrm{Control}}-{\mathrm{OD}}_{\mathrm{Background}}}$$

where *OD*_Treated_ and *OD*_Control_ present the absorbance at 570 nm for groups treated with different samples and PBS buffer, respectively, and *OD*_Background_ is the absorbance of the background group.

### 3.12. Intracellular ROS Scavenging

The ROS scavenging capability of inhibitors was detected by dichlorodihydrofluorescein diacetate (DCFH-DA) assay (Beyotime, Shanghai, China, S0033S). SH-SY5Y cells were added to 96-well plates (8000 cells/well) and incubated for 24 h. Subsequently, 20 μL of a mixed solution (containing 25 μM Aβ_40_ and different concentrations of inhibitors) that had been preincubated for 24 h was added, and the incubation was continued for 24 h. After that, a DCFH-DA fluorescent probe (10 μM dissolved in serum-free medium, 100 μL) was added and the cells were incubated in a dark environment for 30 min. The cell morphology and its fluorescence were observed using a TE2000-U inverted fluorescence microscope (Nikon, Tokyo, Japan).

### 3.13. C. elegans Strain Experiments

Two species of *C. Elegans* were used in the study, which were a wild-type nematode (N2) and a transgenic AD nematode (CL2006). Nematodes were cultured in nematode growth medium using *E. coli* OP50 spread. The nematode growth medium (pH 6.0) contained 1 mM CaCl_2_, 1 mM MgSO_4_, 5 μg/mL cholesterol, 250 mM KH_2_PO_4_, 17 g/L agar, 2.5 g/L peptone, and 3 g/L NaCl.

The ability of inhibitors to target Aβ plaques within nematodes was examined by ThT-Cy5 dual fluorescence assay. For imaging of Aβ plaques in nematodes, adult CL2006 nematodes were picked and fixed in 4% paraformaldehyde at 4 °C for 24 h. Then the nematodes were stained with ThT solution (10 μM) and Cy5-labelled inhibitor solution (50 μg/mL) for 4 h. The stained nematodes were observed using the inverted fluorescence microscope described above.

To investigate the ability of inhibitors to scavenge Aβ plaques in nematodes, CL2006 nematodes at the L4 period were picked and cultured in fresh nematode growth medium containing inhibitor (50 μg/mL, 300 μL). After incubation for 72 h, nematodes were fixed in 4% paraformaldehyde for 24 h and stained with ThT solution (10 μM). The stained nematodes were placed under the inverted fluorescence microscope for observation.

In the nematode longevity assay, L4-period nematodes were picked and transferred to fresh nematode growth medium (50 nematodes/plate) (containing 300 μL of 50 μg/mL inhibitor). The number of surviving nematodes was observed and recorded daily until all nematodes died. Among the criteria for determining nematode death were that the nematodes did not respond to mechanical stimulation by the platinum wire and no head movement of the nematodes was observed. Every 3 days, the surviving nematodes from each group were transferred to fresh nematode growth medium to ensure adequate food for the nematodes.

### 3.14. Statistical Analysis

All data were expressed as mean values ± standard deviation. All statistical analyses were conducted using a one-way analysis of variance (ANOVA) followed by a statistical comparison using a Tukey test, and *p* < 0.05 or less was accepted as statistically significant.

## 4. Conclusions

In this study, a fusion protein, KLVFF-TTR-Pen, derived from TTR, was engineered by incorporating the Aβ-targeting peptide KLVFF and cell-penetrating peptide penetratin (Pen) into TTR. Subsequently, a nanocomposite of KTP and MnO_2_ nanoclusters, denoted as KTP@MnO_2_, was synthesized through a biomineralization process, which was further explored as a multifunctional inhibitor against Alzheimer’s β-amyloid fibrillogenesis. KTP@MnO_2_ demonstrated remarkable efficacy in inhibiting Aβ fibrillization, surpassing the performance of KTP and TTR. The BBB penetration and Aβ-targeting of KTP@MnO_2_ were validated by transwell experiments and fluorescence microscopy. Additionally, KTP@MnO_2_ can scavenge various ROS, including ·OH, ·O_2_^−^, H_2_O_2_, and Aβ-induced ROS, mitigating cellular oxidative damage. KTP@MnO_2_ attenuated Aβ-mediated cytotoxicity and prolonged the lifespan of CL2006 nematodes from 12 d to 19 d by in vivo deposition of Aβ plaques. This work provided a new insight into the development of potential multifunctional amyloid inhibitors based on protein and metal nanoclusters.

## Data Availability

Data are contained within the article and Supplementary Materials.

## Supplementary Materials

The following supporting information can be downloaded at: https://www.mdpi.com/article/10.3390/molecules29061405/s1, Table S1: Lag time (*T*_lag_) of Aβ_40_ aggregation in the absence or presence of different inhibitors; Table S2: The content of the secondary structure of Aβ_40_; Figure S1: Aggregation kinetics of Aβ_40_ incubated with TTR-derived proteins; Figure S2: Construction of pCold II-TTR and pCold II-KTP expression vector and SDS-PAGE of TTR and KTP; Figure S3: Aggregation kinetics of Aβ_40_ incubated with different concentrations of TTR, KTP or KTP@MnO_2_; Figure S4: ITC binding isotherm for the titration of TTR and KTP to Aβ_40_; Figure S5: CD spectra of Aβ_40_ incubated with inhibitors; Figure S6: Linear standard curve of Cy5 fluorescence intensity of TTR, KTP or KTP@MnO_2_ versus their concentrations; Figure S7: In vitro targeting capability of different inhibitors; Figure S8: Scavenging ability of ·OH by different concentrations of inhibitors.
