# Supplementary material for: Composite of KLVFF-Transthyretin-Penetratin and Manganese Dioxide Nanoclusters: A Multifunctional Agent against Alzheimer’s β-Amyloid Fibrillogenesis
Source: Molecules. 2024 Mar 21;29(6):1405. doi: 10.3390/molecules29061405 (PMC10975269; doi:10.3390/molecules29061405)
Supplement: Supplementary file 1 [file molecules-29-01405-s001.zip › molecules-2883486-supplementary.pdf]

# Supporting information

## Composite of KLVFF-Transthyretin-Penetratin and Manganese Dioxide Nanoclusters: A Multifunctional Agent against Alzheimer's β-Amyloid Fibrillogenesis

**Haitao Lan [1,†], Ying Wang [1,†], Wei Liu [2,*], Xiaoyan Dong [1] and Yan Sun [1,*]**

[1] Key Laboratory of Systems Bioengineering and Frontiers Science Center for Synthetic Biology (Ministry of Education), Department of Biochemical Engineering, School of Chemical Engineering and Technology, Tianjin University, Tianjin 300350, China; lanhaitao@tju.edu.cn (H.L.); 1020207109@tju.edu.cn (Y.W.); d_xy@tju.edu.cn (X.D.)

[2] Tianjin Key Laboratory of Radiation Medicine and Molecular Nuclear Medicine, Institute of Radiation Medicine, Chinese Academy of Medical Sciences and Peking Union Medical College, Tianjin 300192, China

* Correspondence: liuwei@irm-cams.ac.cn (W.L.); ysun@tju.edu.cn (Y.S.); Tel./Fax: +86-22-27403389 (Y.S.)

† These authors contributed equally to this work.

**Table S1.** Lag time ($T_{lag}$) of A$\beta_{40}$ aggregation kinetics calculated from Figure S2.

| Sample | $T_{lag}$ (h) |
|---|---|
| Aβ only | 49.8 ± 5.8 |
| Aβ + 10 μg/mL TTR | 58.5 ± 2.1 |
| Aβ + 25 μg/mL TTR | 81.1 ± 3.0 |
| Aβ + 50 μg/mL TTR | 86.5 ± 2.2 |
| Aβ + 10 μg/mL KTP | 16.8 ± 1.5 |
| Aβ + 25 μg/mL KTP | 26.4 ± 2.5 |
| Aβ + 50 μg/mL KTP | — — |
| Aβ + 10 μg/mL KTP @$MnO_2$ | 21.2 ± 3.1 |
| Aβ + 25 μg/mL KTP @$MnO_2$ | 68.1 ± 3.5 |
| Aβ + 50 μg/mL KTP @$MnO_2$ | — — |

**Table S2.** The content of secondary structure of A$\beta_{40}$ incubated with different inhibitor was calculated by the BeStSeL algorithm (http://bestsel.elte.hu/). Others include $3_{10}$-helix, bends, π-helix, β-bridge, and irregular/loop.

| Secondary structure | Aβ only | Aβ only (0 h) | + 50 μg/mL TTR | + 50 μg/mL KTP | + 50 μg/mL KTP@$MnO_2$ |
|---|---|---|---|---|---|
| Helix | 22.4 | 30.3 | 12.8 | 11.3 | 0 |
| Antiparallel β-sheet | 20.5 | 64.8 | 35.5 | 44.9 | 44.5 |
| Parallel β-sheet | 57.1 | 0 | 36.8 | 0 | 0 |
| Turn | 0 | 0 | 15 | 13.2 | 9.7 |
| Others | 0 | 5 | 0 | 30.6 | 45.8 |

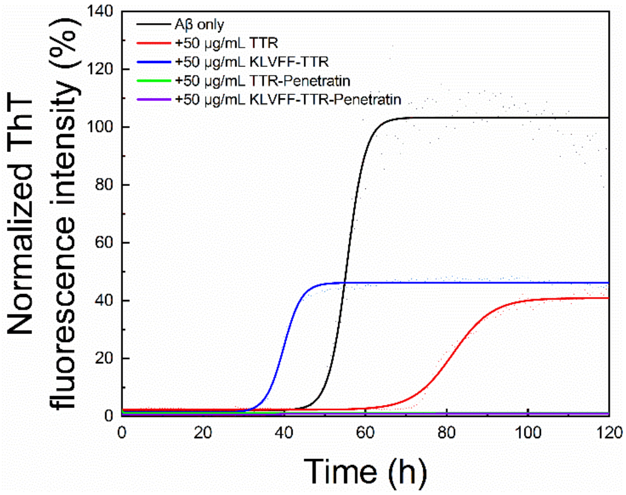


**Figure S1.** Aggregation kinetics of $A\beta_{40}$ incubated with TTR-derived proteins.

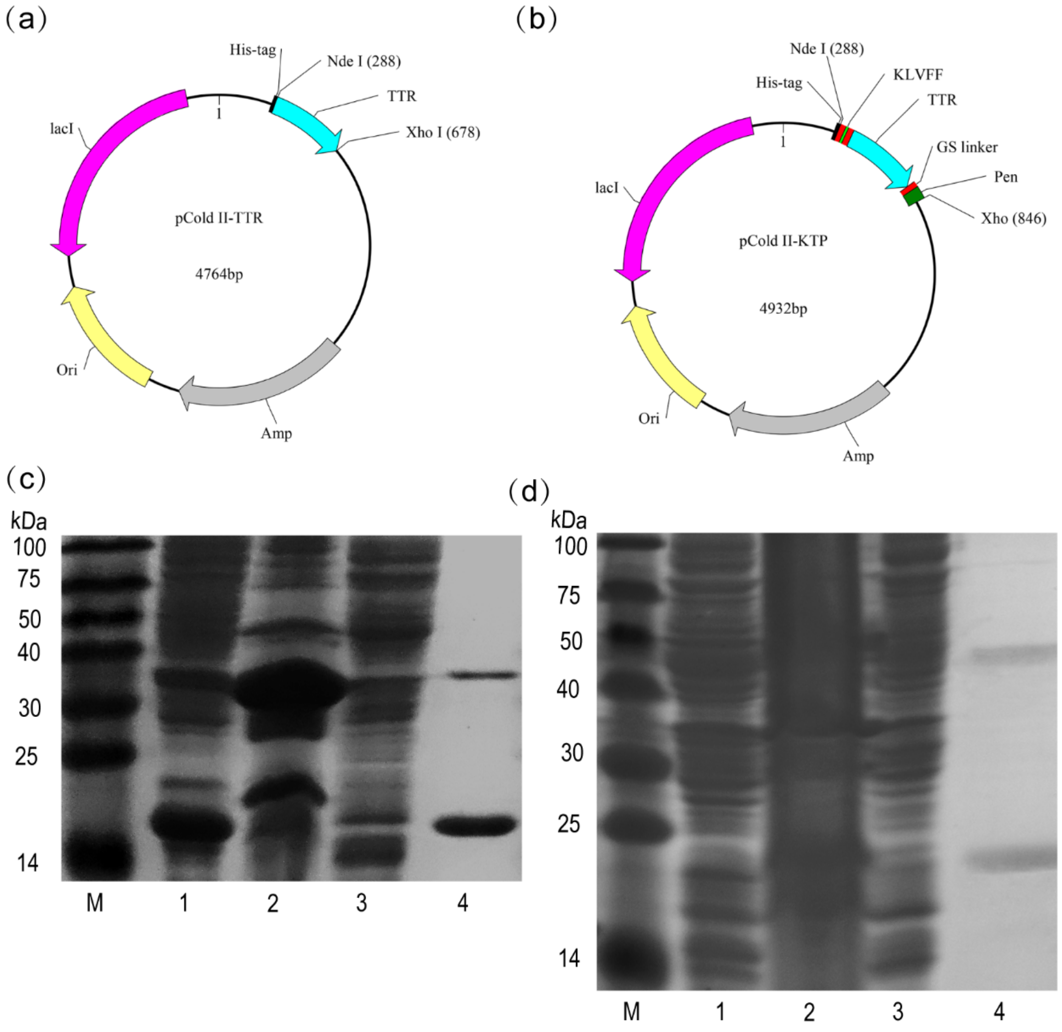


**Figure S2.** (a)-(b) Construction of pCold II-TTR and pCold II-KTP expression vectors. (c) SDS-PAGE of TTR. Lanes: M, protein marker; 1, supernatant of cell lysate; 2, precipitate of cell lysate; 3, washing eluent; 4, purified TTR. (d) SDS-PAGE of KTP. Lanes: M, protein marker; 1, supernatant of cell lysate; 2, precipitate of cell lysate; 3, washing eluent; 4, purified KTP.

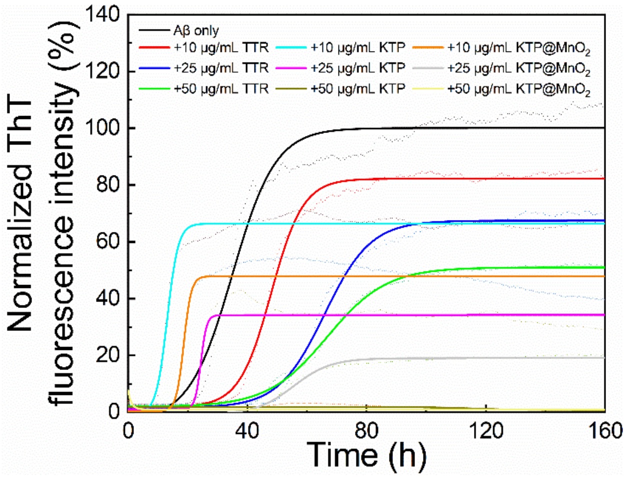


**Figure S3.** Aggregation kinetics of $A\beta_{40}$ incubated with different concentrations of TTR, KTP or KTP@$MnO_2$.

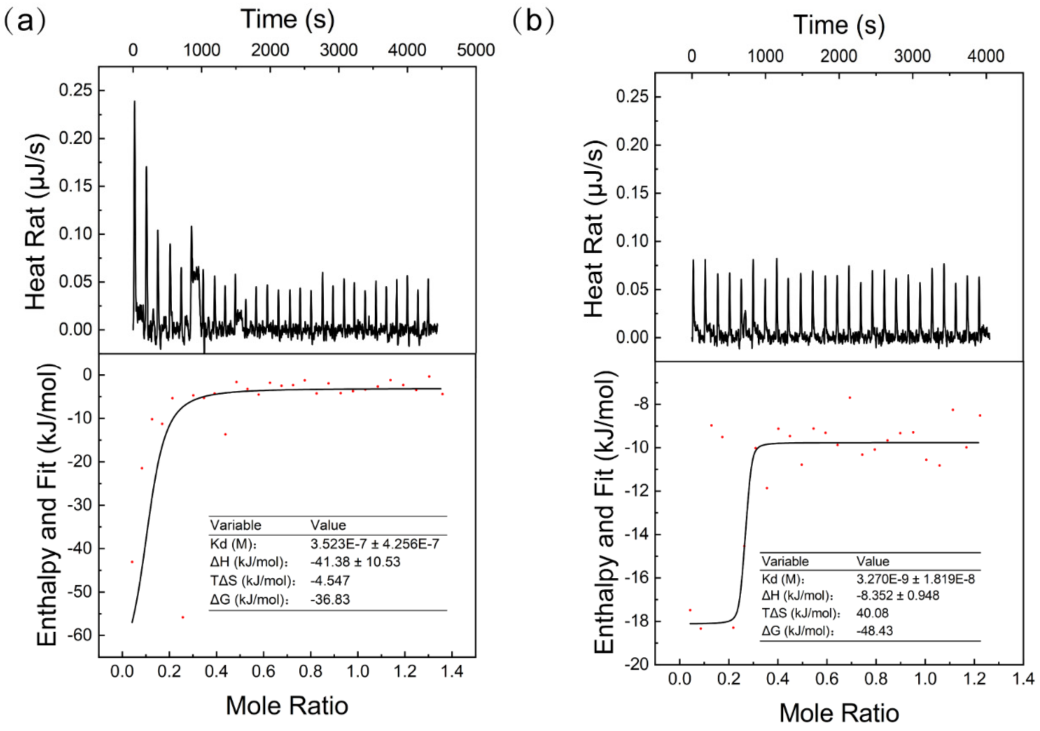


**Figure S4.** ITC binding isotherm for the titration of (a) TTR and (b) KTP to $A\beta_{40}$.

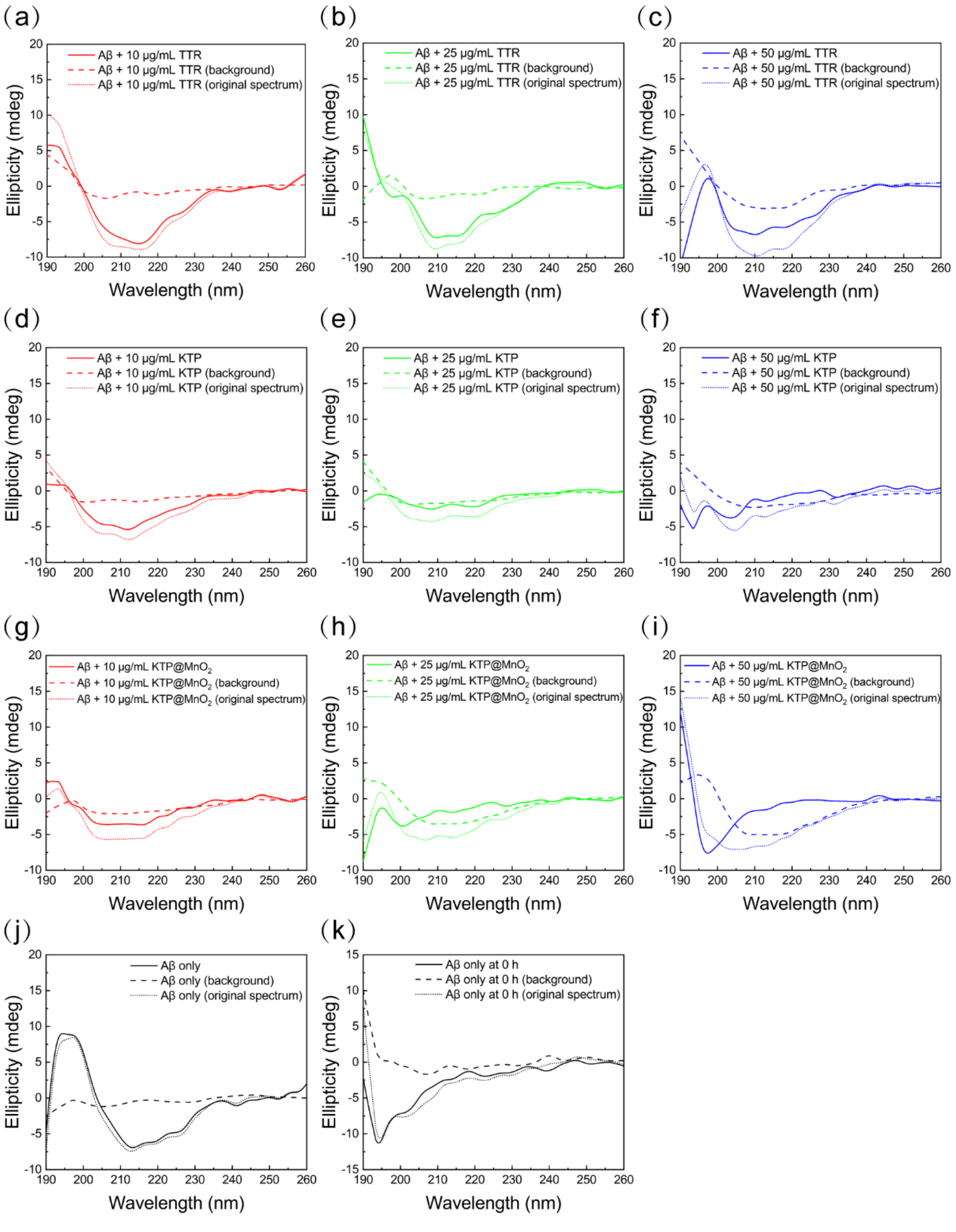


**Figure S5.** CD spectra of Aβ40 incubated with (a) 10 μg/mL TTR, (b) 25 μg/mL TTR, (c) 50 μg/mL TTR, (d) 10 μg/mL KTP, (e) 25 μg/mL KTP, (f) 50 μg/mL KTP, (g) 10 μg/mL KTP@$MnO_2$, (h) 25 μg/mL KTP@$MnO_2$, and (i) 50 μg/mL KTP@$MnO_2$. (j) CD spectra of Aβ40 after incubation. (k) CD spectra of Aβ40 without incubation. (Background is an Aβ-free solution, and the spectrum corresponding to the solid line is the original spectrum minus the background spectrum).

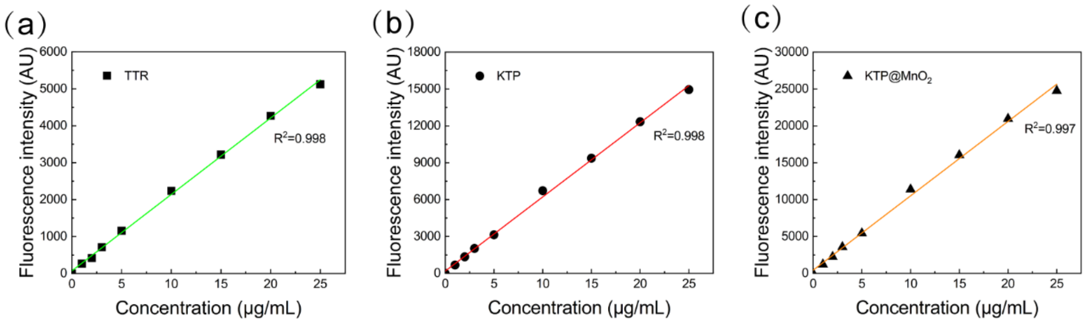


**Figure S6.** Standard curves of Cy5 fluorescence intensity of (a) TTR, (b) KTP, and (c) KTP@$MnO_2$.

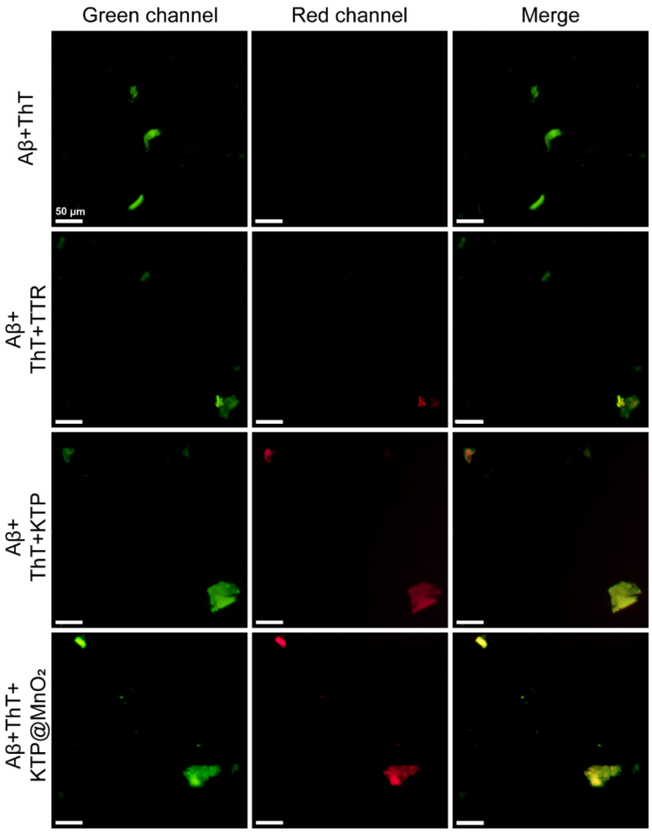


**Figure S7.** *In vitro* targeting capability of different inhibitors. Aβ plaques were stained with Aβ-specific probe ThT (Green channel) and incubated with Cy5-labelled inhibitors (Red channel). Scale bars, 50 μm.

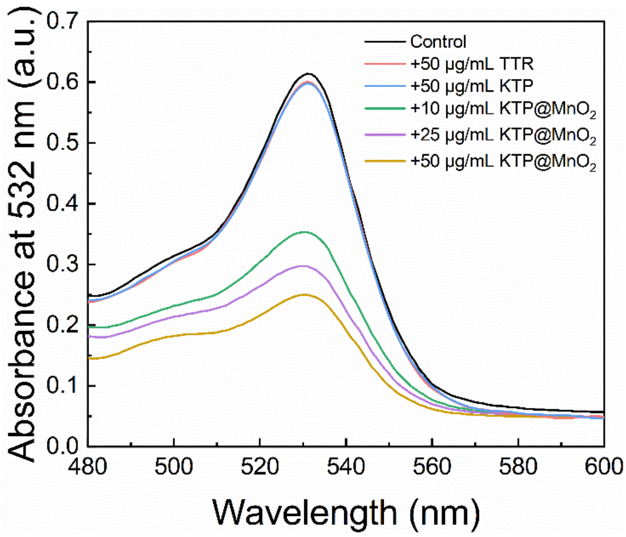


**Figure S8.** ·OH scavenging ability of different inhibitors.
